# Efficient protective activity of a planar catechin analogue against radiation-induced apoptosis in rat thymocytes†

**DOI:** 10.1039/c7ra13111a

**Published:** 2018-03-13

**Authors:** Emiko Sekine-Suzuki, Ikuo Nakanishi, Kohei Imai, Megumi Ueno, Takashi Shimokawa, Ken-ichiro Matsumoto, Kiyoshi Fukuhara

**Affiliations:** Quantitative RedOx Sensing Team (QRST), Department of Basic Medical Sciences for Radiation Damages, National Institute of Radiological Sciences (NIRS), National Institutes for Quantum and Radiological Science and Technology (QST) Inage-ku Chiba 263-8555 Japan nakanishi.ikuo@qst.go.jp +81-43-255-6819 +81-43-206-3131; School of Pharmacy, Showa University Shinagawa-ku Tokyo 142-8555 Japan

## Abstract

About two thirds of biological damage due to low linear energy transfer (LET) radiation, such as X-rays and the plateau region of heavy-ion beams, is known to be caused by the hydroxyl radical (˙OH), the most powerful reactive oxygen species (ROS), generated *via* ionisation and excitation of water molecules. Thus, compounds having an efficient scavenging activity against ROS are expected to exhibit a radioprotective activity. A planar catechin analogue, where an isopropyl fragment was introduced into the catechol ring of (+)-catechin, showed an efficient protective effect against X-ray induced apoptosis in rat thymocytes compared to (+)-catechin. The planar catechin scavenged 2,2-diphenyl-1-picrylhydrazyl radicals (DPPH˙) solubilised in water by β-cyclodextrin about 10-fold faster than (+)-catechin in phosphate buffer (0.1 M, pH 7.4) at 298 K. Furthermore, the experimental log *P* value of the planar catechin (1.22) is reported to be significantly larger than that of (+)-catechin (0.44). The higher radical-scavenging activity and lipophilicity of the planar catechin than those of (+)-catechin may contribute in part to the higher protective activity against X-ray-induced apoptosis in rat thymocytes.

## Abstract 2

A planar catechin analogue showed a significant higher protective activity against X-ray induced apoptosis in rat thymocytes than (+)-catechin.

## Graphical abstract

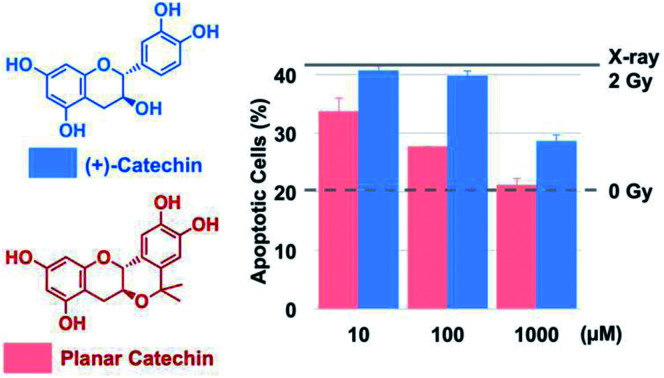

## Introduction

Ionising radiation is one of the effective methods for cancer treatment. To maintain the quality of life (QOL) of cancer patients, the demand for radiotherapy that can maintain the shape of internal organs has been increasing in recent years. However, the adverse effect of radiotherapy on normal tissues adjacent to the tumour cannot be ignored. If the tolerance of normal tissues toward radiation could be improved, higher doses of radiation could be employed.^1^ It is known that about two thirds of the biological damage due to low linear energy transfer (LET) radiation, such as X-rays and the plateau region of heavy-ion beams, is caused by the hydroxyl radical (˙OH), the most powerful reactive oxygen species (ROS), generated *via* ionisation and excitation of water molecules.^2^ Thus, compounds having a scavenging activity against ROS are expected to exhibit a radioprotective activity. Among radical scavengers, amifostine (WR-2721), a thiophosphate, shows a significant radioprotective activity and has been approved by the United States Food and Drug Administration (FDA) for patients undergoing radiotherapy. However, the toxicity of amifostine has greatly restricted its use.^3^

**Fig. 1:**
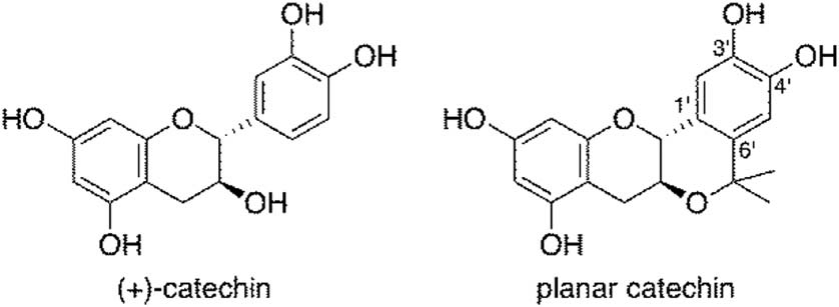
Chemical structures of catechins.

Because natural antioxidants exhibit efficient radical-scavenging activity and little toxicity, they have recently attracted considerable interest as candidates of radioprotectors.^4^ Recently, we have developed a simple and easy method for evaluation of radioprotective activity of chemical compounds using rat thymocytes and demonstrated that natural antioxidants, such as (+)-catechin, resveratrol, caffeic acid and quercetin, exhibit an efficient protective activity against X-ray-induced apoptosis in rat cymocytes.^5^ On the other hand, we have investigated chemical modifications of natural antioxidants to improve their radical-scavenging activity. Introduction of methyl groups at positions *ortho* and/or *para* to the phenolic hydroxyl group in antioxidants resulted in a significant enhancement of their radical-scavenging activity.^6^ Among the chemically modified antioxidants, a planar catechin analogue (Fig. 1), where an isopropyl fragment was introduced into (+)-catechin, exhibited 5-fold more potent radical-scavenging activity compared to (+)-catechin in acetonitrile and a protective effect against Fenton reaction induced DNA damage with no pro-oxidant effect.^7^ We also reported that the radical-scavenging reaction of (+)-catechin in acetonitrile proceeds *via* an electron transfer from (+)-catechin to the radical to produce the corresponding radical cation of (+)-catechin, followed by proton transfer.^8^ The electron-donating isopropyl group at the C6′ position (Fig. 1) in the planar catechin may stabilise the radical cation intermediate, resulting in such an enhanced radical-scavenging activity. In fact, the oxidation potential of the planar catechin is reported to be significantly lower than that of (+)-catechin.^7*c*^ Furthermore, Bernini *et al.* reported that a methylated planar catechin derivative is much more easily oxidized than a methylated (+)-catechin derivative.^9^ The planar catechin also shows several biological activities, such as remarkable α-glucosidase inhibition, and anti-virus and anti-tumour activities.^7*d*,10^ We report herein that the enhanced radioprotective activity of the planar catechin against X-ray induced apoptosis in rat thymocytes compared to that of (+)-catechin. The biological data using rat thymocytes together with chemical data, such as radical-scavenging rates of the catechins as well as their lipophilicity provide fundamental and valuable information to develop effective radioprotective agents without toxicity.

## Experimental

### Materials

(+)-Catechin was purchased from Sigma and purified by column chromatography on silica gel (7 : 3 : 1 toluene–acetone–methanol). The planar catechin was synthesised according to the procedure reported in the literatures.^7*a*,*d*,*e*^ 2,2-Diphenyl-1-picrylhydrazyl radical (DPPH˙) was commercially obtained from Wako Pure Chemical Ind. Ltd., Japan. β-Cyclodextrin (β-CD) was purchased from Tokyo Chemical Industry Co., Ltd., Japan, recrystallised from water and dried under vaccum at 313 K. The water used in this study was freshly prepared with a Milli-Q system (Millipore Direct-Q UV3). DPPH˙ was solubilised in water by β-CD according to the procedure reported in the literatures.^11^

### Animals

All animal experiments were performed in National Institute of Radiological Sciences (NIRS), conformed to institutional guidelines, and were approved by the Institutional Animal Care and Use Committee of NIRS. Male Wister-MS rats were obtained at 8 weeks of age from Japan SLC, Inc. The rats were housed in a temperature- and humidity-controlled room at 23 ± 1 °C and 55 ± 5%, respectively, and maintained for on a 12 h light–dark cycle. The rats were kept two per cage. They received acidified water and diet (MB-1, Funabashi Farm Co., Japan) ad libitum during the experimental period.

### Preparation of thymocyte samples

The thymus from 10–15 week old rats was washed in cold Dulbecco's phosphate-buffered saline (Sigma) to remove extra fat and placed in Roswell Park Memorial Institute medium (RPMI 1640) (Sigma) supplemented with 10% fetal bovine serum (FBS). The thymus was cut and squeezed to thymocytes by using tweezers. These cells were suspended through nyon mesh and then centrifuged at 1500 rpm for 5 min at 4 °C with centrifuge (SAKUMA M-160-IV). The cell pellet was resuspended in PRMI 1640 with 10% FBS and the number of cells were counted under a microscope (OLYMPUS IX-70). The cells were seeded in 48 wells plates at a density of 5.0 × 10^5^ cells per well on ice.

### X-ray irradiation

Cells were irradiated with X-rays using a Shimadzu Pantak HF-320 at a dose rate of 1.2 Gy min^−1^. (effective energy 80 keV, tube voltage 200 kV, tube current 20 mA, 0.5 mm aluminum + 0.5 mm copper filter). The radiation dose was determined using a dose meter. The typical microscopic images of the thymocytes before and after X-ray irradiation are shown in our previous paper.^5*a*^

### Flow cytometry analysis

After irradiation, the cells were incubated for 4 h at 37 °C with 5% CO_2_. After incubation, the size and number of thymocytes were measured on a flow cytometer FACS Calibur (Becton Dickinson Company). At least 10 000 cells were measured for each sample, and the data were analysed with BD CellQuest Pro software (Becton Dickinson Company).

### Spectral and kinetic measurements

The rates of DPPH˙-scavenging reactions by catechins in phosphate buffer (0.1 M, pH 7.4) were determined by monitoring the absorbance change at 527 nm due to DPPH˙ after mixing of β-CD-solubilised DPPH˙ (DPPH˙/β-CD) in water (Milli-Q) with a phosphate buffer solution (0.2 M, pH 7.4) containing catechins at a volumetric ratio of 1 : 1 using a stopped-flow technique on a UNISOKU RSP-1000-02NM spectrophotometer at 298 K. Therefore, the final concentration of the phosphate buffer is 0.1 M. The *k*_obs_ values were determined by a least-square curve fit using an Apple MacBook Pro personal computer. The first-order plots of ln(*A* − *A*_∞_) *vs.* time (*A* and *A*_∞_ are denoted as the absorbance at the reaction time and the final absorbance, respectively) were linear until three or more half-lives with the correlation coefficient *ρ* > 0.999.

## Results and discussion

### Protective activity of catechins against X-ray induced apoptosis in rat thymocytes

**Fig. 2:**
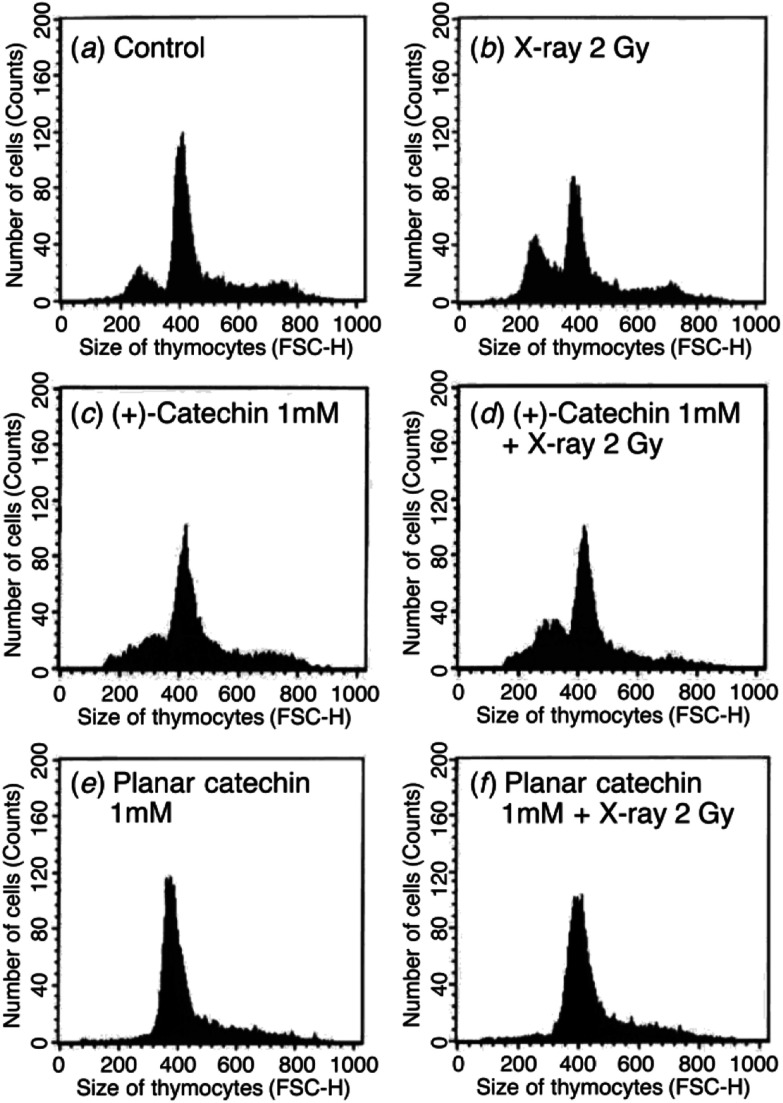
Flow cytometer analysis of the rat thymocytes. (a) Cells were incubated in the presence of 0.1% DMSO for 4 h. (b) Cells were incubated in the presence of 0.1% DMSO for 4 h after 2 Gy X-ray irradiation. (c) Cells were incubated in the presence of 1 mM (+)-catechin and 0.1% DMSO as a cosolvent for 4 h. (d) Cells were incubated in the presence of 1 mM (+)-catechin and 0.1% DMSO as a cosolvent for 4 h after 2 Gy X-ray irradiation. (e) Cells were incubated in the presence of 1 mM planar catechin and 0.1% DMSO as a cosolvent for 4 h. (f) Cells were incubated in the presence of 1 mM planar catechin and 0.1% DMSO as a cosolvent for 4 h after 2 Gy X-ray irradiation.

**Fig. 3:**
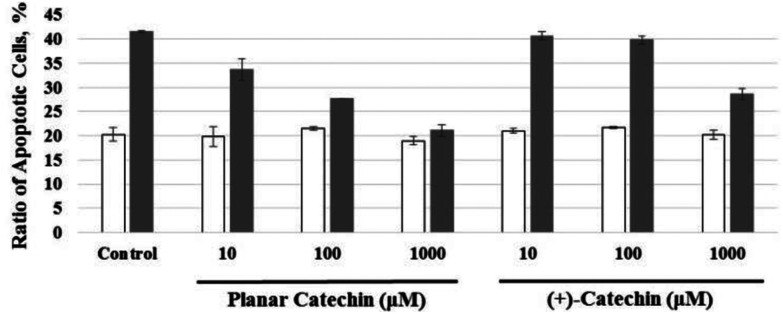
Effects of various concentrations of the planar catechin and (+)-catechin on apoptosis in rat thymocytes without (white bars) and with (gray bars) 2 Gy X-ray irradiation.

The radioprotective activity of two catechins in Fig. 1 was examined using the assessment method reported in the leteratures.^5^ The radioprotective activity of chemical compounds is estimated by quantifying the frequency of apoptosis induced by radiation in rat thymocytes. Thymocytes from rats were placed in cell culture medium (RPMI 1640) supplemented with 10% FBS. The catechins were dissolved in DMSO as a cosolvent and applied to the cell suspensions (1, 10, 100 or 1000 μM). The thymocytes were pretreated with either DMSO 0.1% or the catechins with DMSO 0.1% as a cosolvent. After 2 Gy X-ray irradiation, the cells were incubated for 4 h at 37 °C with 5% CO_2_. After incubation, the size and number of thymocytes were measured by the flow cytometer. Fig. 2 shows the distribution of the cell size after X-ray irradiation (0 or 2 Gy) with or without catechins (1 mM) (see the Fig. S1, ESI† for other concentrations). The control cells show a sharp peak at around 400 with a small peak at around 250 (Fig. 2a), while 2 Gy X-ray irradiation resulted in the decrease of the peak height at around 400 accompanied by an increase in a peak at around 250 (Fig. 2b). Fig. 2d and f shows the thymocytes treated with 1 mM (+)-catechin and planar catechin, respectively, before X-ray irradiation, where the decrease in the peak at around 400 is significantly inhibited, suggesting that these catechins protects the rat thymocytes. Fig. 2c and e show that 1 mM catechins themselves did not induce apoptosis in the thymocytes. The white and gray bars in Fig. 3 show percentages of the apoptotic cells without and with X-ray irradiation, respectively. When the white bar in the presence of a compound is larger than that in its absence (control), the compound shows toxicity to the thymocytes without X-ray irradiation. Thus, no toxicity was observed by both (+)-catechin and the planar catechin (Fig. 3). On the other hand, a smallar gray bar in the presence of a compound indicates that the compound shows a radioprotective activity. In the control sample, 2 Gy of X-ray irradiation induced apoptosis in about 40% of the thymocytes, while about 20% of the thymocytes died without X-ray irradiation (Fig. 3). The apoptosis induced by X-ray irradiation was efficiently suppressed by the planar catechin pretreatment in a concentration-dependent manner as shown in Fig. 3. The ratio-modification factor (RMF) is defined as the ratio of apoptotic cells with chemicals divided by the ratio of apoptotic cells without chemicals in order to quantify the radioprotective efficiency of the chemicals. The lower RMF values of the compounds are, the higher the radioprotective activity becomes. In this study, 10, 100 and 1000 μM planar catechin exhibited 0.7, 0.1 and 0 of RMF, respectively (Table 1). In contrast, 10, 100 and 1000 μM (+)-catechin showed 0.9, 0.9 and 0.4 RMF, respectively (Table 1). Thus, the planar catechin showed significantly more efficient radioprotective activity against X-ray induced apoptosis in rat thymocytes compared to (+)-catechin.

**Table 1:** RMF values of catechins

Concentration/μM	Ratio of dead cells/%		RMF protectivity
	No IR	X-ray 2 Gy	
Control	20.25 ± 1.37	41.51 ± 0.11	1.0 ± 0.01
Planar catechin 10	19.80 ± 2.11	33.66 ± 2.33	0.7 ± 0.03
100	21.45 ± 0.37	27.66 ± 0.05	0.1 ± 0.02
1000	18.98 ± 0.88	21.09 ± 1.22	0 ± 0.03
(+)-Catechin 10	21.01 ± 0.42	40.65 ± 0.80	0.9 ± 0.04
100	21.63 ± 0.17	39.76 ± 0.86	0.9 ± 0.04
1000	20.23 ± 0.93	28.62 ± 1.05	0.4 ± 0.05

### Radical-scavenging activity of the planar catechin in phosphate buffer

**Fig. 4:**
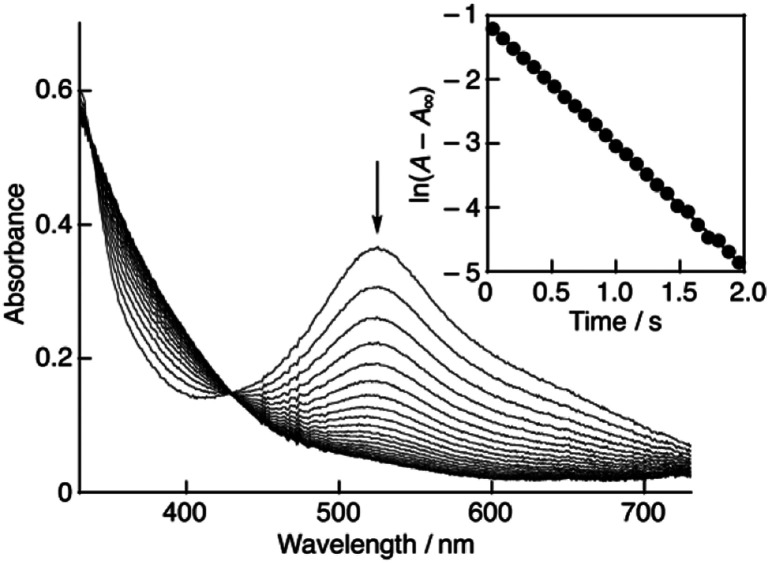
Spectral change (interval: 20 ms) observed during the reaction of (+)-catechin (1.9 × 10^−3^ M) with DPPH˙/β-CD (3.2 × 10^−5^ M) in phosphate buffer (0.1 M, pH 7.4) at 298 K. Inset: the pseud-first-order plot based on the absorbance at 527 nm.

**Fig. 5:**
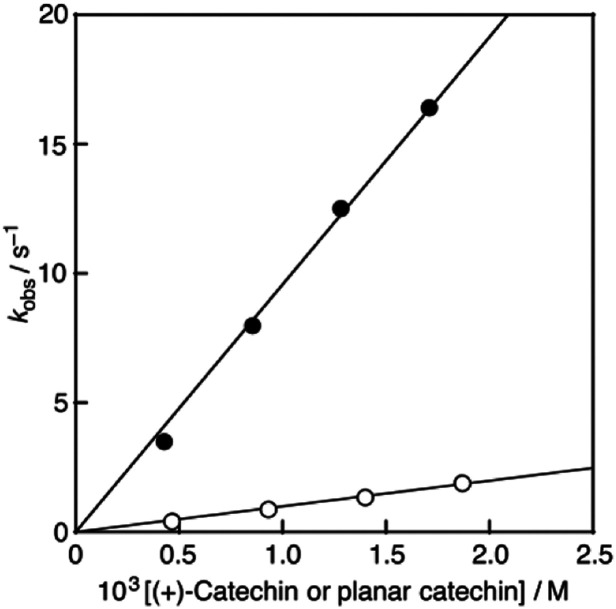
Plots of *k*_obs_*vs.* [(+)-catechin] (open circles) and [planar catechin] (closed circles).

Recently, we have reported that 2,2-diphenyl-1-picrylhydrazyl radical (DPPH˙), a stable radial frequently used as a reactivity model of ROS,^12–16^ could successfully be solubilised in water using β-cyclodextrin (β-CD).^11^ This enables us to evaluate the radical-scavenging activity of antioxidants in buffer solutions under physiological conditions. Thus, the radical-scavenging activity of (+)-catechin and the planar catechin was investigated using β-CD-solubilised DPPH˙ (DPPH˙/β-CD) in phosphate buffer (0.1 M, pH 7.4). Upon mixing of (+)-catechin in phosphate buffer solution (0.2 M, pH 7.4) with a water (Milli-Q) solution of DPPH˙/β-CD at a volumetric ration of 1 : 1 on a stopped-flow spectrophotometer, the absorption band at 527 nm due to DPPH˙/β-CD immediately decreased with a clear isosbestic point at 429 nm as shown in Fig. 4. The decay of the absorbance at 527 nm obeyed pseudo-first-order kinetics, when the concentration of (+)-catechin was higher than a 10-fold excess of the DPPH˙/β-CD concentration (inset of Fig. 4). The pseudo-first-order rate constant (*k*_obs_) linearly increased as the (+)-catechin concentration ([(+)-catechin]) increased (Fig. 5). Based on the slope of the linear plot, the second-order-rate constant (*k*) for the reaction between (+)-catechin and DPPH˙/β-CD was determined in phosphate buffer (0.1 M, pH 7.4) at 298 K to be 9.9 × 10^2^ M^−1^ s^−1^. The *k* value for the reaction between the planar catechin and DPPH˙/β-CD was also determined in the same manner to be 9.6 × 10^3^ M^−1^ s^−1^, which is almost 10-fold larger compared to the case of (+)-catechin. Thus, the planar catechin exhibits a much stronger radical-scavenging activity than (+)-catechin in phosphate buffer (0.1 M, pH 7.4).

Moreover, the experimental log *P* value of the planar catechin (1.22) is reported to be significantly larger than that of (+)-catechin (0.44),^17^ indicating that the lipophilicity as well as membrane permeability of the planar catechin is higher compared to (+)-catechin.

## Conclusions

The planar catechin exhibited a significantly more efficient radioprotective activity against the radiation-induced apoptosis in rat thymocytes compared to (+)-catechin. The higher radical-scavenging activity as well as lipophilicity of the planar catechin may contribute in part to such a higher radioprotective activity than (+)-catechin. To sophisticate the radiation cancer therapy, it is of considerable importance to develop radioprotectors without adverse side effects for normal tissues around tumours. The results obtained in this study suggest that the planar catechin may be one of the promising lead compounds that can be used in combination with radiation cancer therapy. Further experiments are underway to investigate the structure–activity relationships for the effective radioprotective agents using natural and synthetic antioxidants.

## Conflicts of interest

There are no conflicts to declare.

## Supplementary Material

RA-008-C7RA13111A-s001
