# Supplementary material for: Efficient protective activity of a planar catechin analogue against radiation-induced apoptosis in rat thymocytes
Source: RSC Adv. 2018 Mar 13;8(19):10158–62. doi: 10.1039/c7ra13111a (PMC9078822; doi:10.1039/c7ra13111a)
Supplement: RA-008-C7RA13111A-s001 [file RA-008-C7RA13111A-s001.pdf]

Electronic Supplementary Material (ESI) for RSC Advances.
This journal is © The Royal Society of Chemistry 2018

Electronic Supplementary Information (ESI)

Efficient protective activity of a planar cartechin analogue against radiation-induced apoptosis in rat thymocytes

Emiko Sekine-Suzuki,[a] Ikuo Nakanishi,*[a] Kohei Imai,[ab] Megumi Ueno,[a] Takashi Shimokawa,*[a] Ken-ichiro Matsumoto[a] and Kiyoshi Fukuhara,*[b]

[a] *Quantitative RedOx Sensing Team (QRST), Department of Basic Medical Sciences for Radiation Damages, National Institute of Radiological Sciences (NIRS), National Institutes for Quantum and Radiological Science and Technology (QST), Inage-ku, Chiba 263-8555, Japan.*

[b] *School of Pharmacy, Showa University, Shinagawa-ku, Tokyo 142-8555, Japan.*

E-mail: nakanishi.ikuo@qst.go.jp, shimokawa.takashi@qst.go.jp, fukuhara@pharm.showa-u.ac.jp

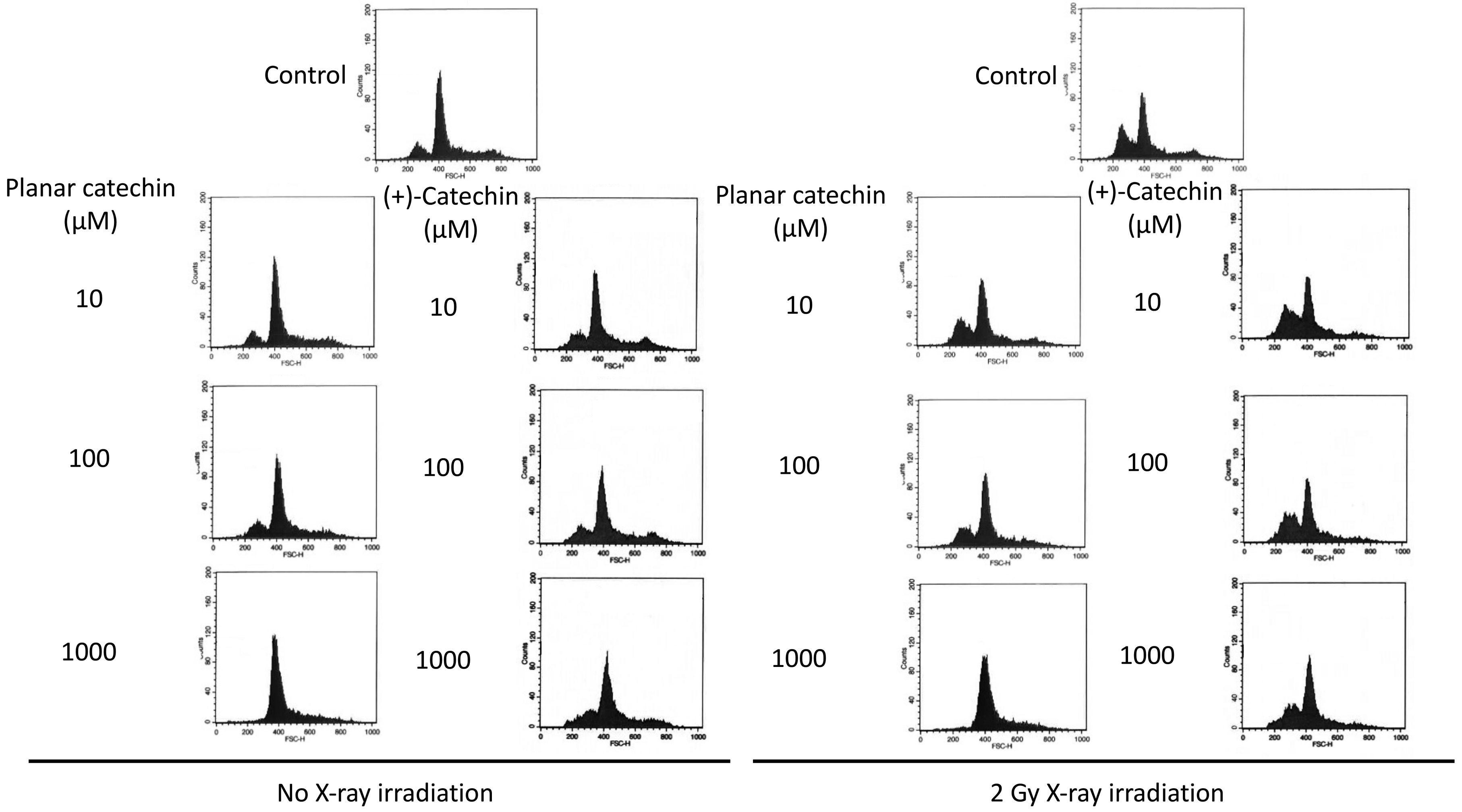

Fig. S1 Flow cytometer analysis of the rat thymocytes. Cells were incubated in the presence of 0.1% DMSO with or without catechins (0, 10, 100 and 1000 μM) for 4h after X-ray irradiation (0 or 2 Gy).
